# A dataset of occurrence of wild bees and their interaction with foraging plants along a livestock grazing gradient of northern Tanzania

**DOI:** 10.1016/j.dib.2023.109181

**Published:** 2023-04-24

**Authors:** Julius V. Lasway, Ingolf Steffan-Dewenter, Rudolf Mremi, Neema R. Kinabo, John J. Sanya, Oliver C. Nyakunga, Emanuel H. Martin, Connal Eardley, Alain Pauly, Marcell K. Peters, Henry K. Njovu

**Affiliations:** a Department of Animal Ecology and Tropical Biology, Biocenter, University of Würzburg, Am Hubland, 97074 Würzburg, Germany; b Department of Wildlife Management, College of African Wildlife Management, Mweka, 214 Mweka Chini HM 25216 Kibosho Mashariki, Moshi, Tanzania; c School for Field Studies: Center for Wildlife Management Studies, P.O. Box 314, Karatu, Tanzania; d Unit for Environmental Sciences and Management, North-West University, Potchefstroom 2520, South Africa; e Royal Belgian Institute of Natural Sciences (RBINS), O.D. Taxonomy & Phylogeny, Rue Vautier 29, B-1000 Brussels, Belgium

**Keywords:** Bee-plant interaction network, Hand net, Livestock grazing intensity, Pan trap, Plant forage resource, Species richness, Standardized random walk

## Abstract

A dataset describing the occurrence of wild bees and their interaction with forage plants along livestock grazing gradient is critical in understanding bee-plant interaction networks and in developing conservation plans to ensure ecosystem services in human-modified landscapes. Despite this need, bee-plant datasets are scarce in Africa, and Tanzania is no exception. Therefore, in this article, we present a dataset of wild bee species richness, occurrence, and distribution collected across sites with different levels of livestock grazing intensity and forage resources thereby.

The data presented in this paper supports a research article by Lasway et al., 2022 describing the effects of grazing intensity on East African bee assemblages. The paper presents primary data on bee species, collection method, date of collection, bee family, identifier, plant forage resource, forage plant life form, forage plant family, location (GPS coordinates), grazing intensity category, mean annual temperature (°C), and elevation (m asl). The data were collected intermittently between August 2018 and March 2020 from 24 study sites distributed along three levels of livestock grazing intensity with eight replicates for each: low, moderate, and high livestock grazing intensity. In each study site, two 50 × 50 m study plots were set from which bees and floral resources were sampled and quantified. The two plots were placed in a way to capture the overall structural heterogeneity of the respective habitat by placing the two plots in contrasting microhabitats where possible. For example, in moderately livestock-grazed habitats, plots were placed on sites with and without tree or shrub cover to ensure representativeness. This paper presents a dataset comprising 2,691 bee individuals from 183 species representing 55 genera of the five bee families: Halictidae (74), Apidae (63), Megachilidae (40), Andrenidae (5), and Colletidae (1). In addition, the dataset comprises 112 species of flowering plants that were identified as potential forage resources for bees. This paper supplements rare but critical data on bee pollinators in Northern Tanzania and advances our knowledge of the potential drivers of bee-pollinator whose populations diversity are declining globally. The dataset will also promote collaborations among researchers who would wish to combine and extend their data for further analysis to gain a broader understanding of the phenomenon on a larger spatial scale.

**Specifications Table**

**Table:** 

Subject	Biological sciences
Specific subject area	Pollination ecology and pollinator diversity
Type of data	Table
How the data were acquired	The data were acquired via plot-based field survey. This involved capturing bees using pan trap and hand net in standardized random walks. Data were then entered into computer Microsoft Excel 2016 before being made publicly available on the Fig share data repository.
Data format	Raw
Description of data collection	Data on bees were collected in three different levels of livestock grazing intensity (GI) using two standardized methods (pan trap and random walks with a hand net). Sampling was restricted to days with no or very little rain and low wind speed. Other variables in the dataset such as mean annual temperature (MAT) was obtained using DS1921G Thermochron iButton data logger positioned at 2 m height on the branch of a tree. Information on forage plant species, plant life form, and bee families were obtained from published sources such as databases and flora books.
Data source location	• City/Town/Region: Kilimanjaro, Arusha, and Manyara regions • Country: Tanzania
Data accessibility	Repository name: Figshare Data identification number: 10.6084/m9.figshare.21550545.v3 Direct URL to data: https://doi.org/10.6084/m9.figshare.21550545.v3
Related research article	Lasway, J. V., Steffan-Dewenter, I., Njovu, H. K., Kinabo, N. R., Eardley, C., Pauly, A., & Peters, M. K. (2022). Positive effects of low grazing intensity on East African bee assemblages mediated by increases in floral resources. *Biological Conservation, 267*, 109490. https://doi.org/10.1016/j.biocon.2022.109490

## Value of the Data

• Despite being used in the analysis of the research article by Lasway *et al.* [1], the dataset can also be used as a basis for decision making by policymakers, ecologists, and researchers interested in linking regional and global datasets to draw major conclusions.
• Using data on bees and associated forage resources [2], ecologists can better understand bee-plant interaction networks under which generalist and specialist bee foragers can be identified.
• To the best of our knowledge, this study presents the largest dataset of bee pollinators in northern Tanzania following that of Lasway *et al.* [3]. Therefore, this dataset serves as a baseline dataset necessary for comparative and/or future studies focusing on the effects of livestock grazing on bee pollinator diversity.
• This dataset provides insight into bees' adaptations to the local environment through bee-plant interaction networks in spatially explicit varying degrees of grazing intensity and temporal scales. Consequently, the dataset will enhance local understanding of important factors and variables for the conservation of the world's pollinators.
• The dataset can also promote collaborations among researchers who would wish to combine and extend their data for further analysis to gain a broader understanding of the phenomenon on a large spatial scale.
• Data collected will contribute to the existing global dataset of bees which is critical in understanding the spatial distribution of various wild bee species.

## 1 Objective

One dataset detailing the occurrence of bees across habitats with varying intensities of agricultural and livestock grazing activities was published by Lasway *et al*. [3]. The present dataset was developed to analyze the status and trends of bee pollinator assemblages and their interactions with plant communities in the Afrotropical region of northern Tanzania. Further to that, the dataset was used to analyze the effects of grazing intensity on East African bee assemblages mediated by changes in floral resources [1]. In that regard, the dataset can improve and increase the visibility of the published research article and increase the integrity and reproducibility of our research.

## 2 Data Description

This paper presents a dataset comprised of 2,691 bee individuals sorted into 183 species representing 55 genera found in five bee families: Halictidae (74), Apidae (63), Megachilidae (40), Andrenidae (5), and Colletidae (1) [1,2]. The dataset also comprises 112 species of flowering plants, their families, and life forms (i.e. herb (annual/perennial), shrub, or tree). These plants were identified as potential forage resources for bees. In addition to that, the dataset consists of other variables such as mean annual temperature (in degrees Celsius,°C) which was obtained using DS1921G Thermochron iButton data logger (± 0.5°C resolution; Maxim Integrated Products, USA). The logger was placed at 2 m height above the ground (on a branch of a shrub or a tree) to record the ambient temperature. This variable is important as reference information to record the climate during the collection period. The data also comprises the method used for collecting bees, date of collection and identifier (the person who identified the bee specimen), location (GPS coordinates), grazing intensity (low, moderate, or high livestock grazing intensity), and elevation (m asl) that was obtained by Garmin GPSMAP 64s GPS receiver (USA) and recorded with the accuracy of ± 3 m. This dataset is part of the three-year Bee-Pollinator Monitoring Project, Tanzania (2017 – 2020).

## 3 Experimental Design, Materials and Methods

### 3.1 Bee Sampling

We collected data in 24 replicate study sites (eight in each livestock grazing intensity: low; moderate; and high) in the savannah lowlands of Mt. Kilimanjaro, Mt. Meru, and the areas of Tarangire National Park in Kilimanjaro, Arusha, and Manyara administrative regions, respectively. The study regions are dominated by pastoral communities with different intensities of livestock grazing [1]. The grazing intensity characterization was done based on (i) the level of protection (protected versus unprotected lands) and (ii) the visual inspection of on-site signs of obvious grazing like shortened tufts of grass and grass percentage cover [1]. Low grazing intensity was always placed in the protected areas with shortened tufts of grass between 25 to 125 cm height covering a large part of the ground, 50-75%. Moderate grazing intensity sites were placed outside protected areas where tufts of grasses were not exceeding 50 cm with grass cover between 25-50%. Similar to moderate grazing intensity, high livestock grazing intensity sites were placed outside protected areas with tufts of grass less than 25 cm, with ground cover less than 25%. The study region is characterized by uniform climatic conditions with volcanic soil originating from the volcanic activities of Mt. Meru and Mt. Kilimanjaro [1]. Meaning that the vegetation height did not differ significantly due to microclimatic conditions and soil characteristics.

Two standardized sampling methods, UV-reflecting colored pan traps and random walks with hand nets were employed in the study area to sample bees [4,5]. Four pan trap clusters (each with white, yellow, and blue UV-reflecting colors) were passively used to collect bees [6]. Clustered pan traps were installed at different heights to maximize the trapability rate of bees flying at different heights: at the shrub and herbaceous layer levels (120 cm and 35 cm above the ground, respectively). A drop of odorless liquid soap per approximately 1 L was used to break the surface tension of water so that bees landing on the pan trap were more likely to be captured. Pan traps were left in the field for 48 hours to collect bees [1,4]. Bees collected were temporarily preserved in 70% ethanol before being pinned and identified to species and morpho species levels [1,3,4].

Besides pan trap sampling, standardized random walks with hand nets were also employed to sample bees. This method involves slow pace random walks within plots using a hand net to collect bees foraging on flowers. Using this method, we collected bees for 2 hours on each study site (1 hour per each 50 × 50 m study plot), excluding processing (handling and recording time). Random walks were conducted anytime between 9:00 am and 5:00 pm when bees are expected to be highly active [7]. Random walks sampling were restricted to days with no or very little rainfall and low wind speed because bees are less active on days of heavy rains and high wind speed. Both pan trapping and hand net random walk sampling were conducted in three main seasons of the year: dry season, long and short rainy season. The pan trap sampling effort summed up to 3,456 hours per study site and 82,944 hours for the entire study, while sweep netting summed up to 6 hours of active bee collection for each study site and 144 hours for the whole study. All bees were identified following the nomenclatural system established by Michener [8] with exception of the Halictidae family which followed the taxonomy of Eardley *et al*. [9].

### 3.2 Bee Forage Resources Sampling

Plant species used by bees were recorded when bees were observed walking or landing on the bloom of flowers, not just flying over it. All bee-visited flowering plants during random walks were sampled and identified to species level following the nomenclature system established by Plants of the World Online [10]. Flowering plants that could not be identified in the field, were taken to the National Herbarium of Tanzania located in the Arusha region for morphological identification.

## Ethics Statements

This study involved no experimentation on humans or vertebrate animals. However, the study complied with the Tanzania national guidelines for handling animals. The research clearance and permits were obtained from Commission for Science and Technology (COSTECH) and Tanzania National Parks (TANAPA) with permit number: No:2019-631-NA-2019-235, TNP/HQ/C.10/13.

## CRediT Author Statement

**Ingolf Steffan-Dewenter, Marcell K. Peters** and **Henry K. Njovu:** Conceptualization, Methodology; **Julius V. Lasway, Connal Eardley, Alain Pauly, Rudolf Mremi, John J. Sanya** and **Emanuel H. Martin:** Data curation; **Henry K. Njovu:** Funding acquisition; **Julius V. Lasway:** Investigation; **Henry K. Njovu, Oliver C. Nyakunga, Emanuel H. Martin** and **Neema R. Kinabo:** Project administration; **Ingolf Steffan-Dewenter** and **Marcell K. Peters:** Supervision; **Julius V. Lasway:** Writing original draft; **All authors:** Writing – review & editing.

## Declaration of Competing Interest

The authors declare that they have no known competing financial interests or personal relationships that could have appeared to influence the work reported in this paper.

## Data Availability

Dataset of wild bees and their forage resources along livestock grazing gradient of northern Tanzania (Original data) (figshare).
